# Supplementary material for: Personal and Organizational Factors as Predictors of Life Satisfaction Among Older Adults in Long-Term Care Settings
Source: Healthcare (Basel). 2025 Feb 2;13(3):306. doi: 10.3390/healthcare13030306 (PMC11817048; doi:10.3390/healthcare13030306)
Supplement: Supplementary file 1 [file healthcare-13-00306-s001.zip › healthcare-3413209-supplementary.pdf]

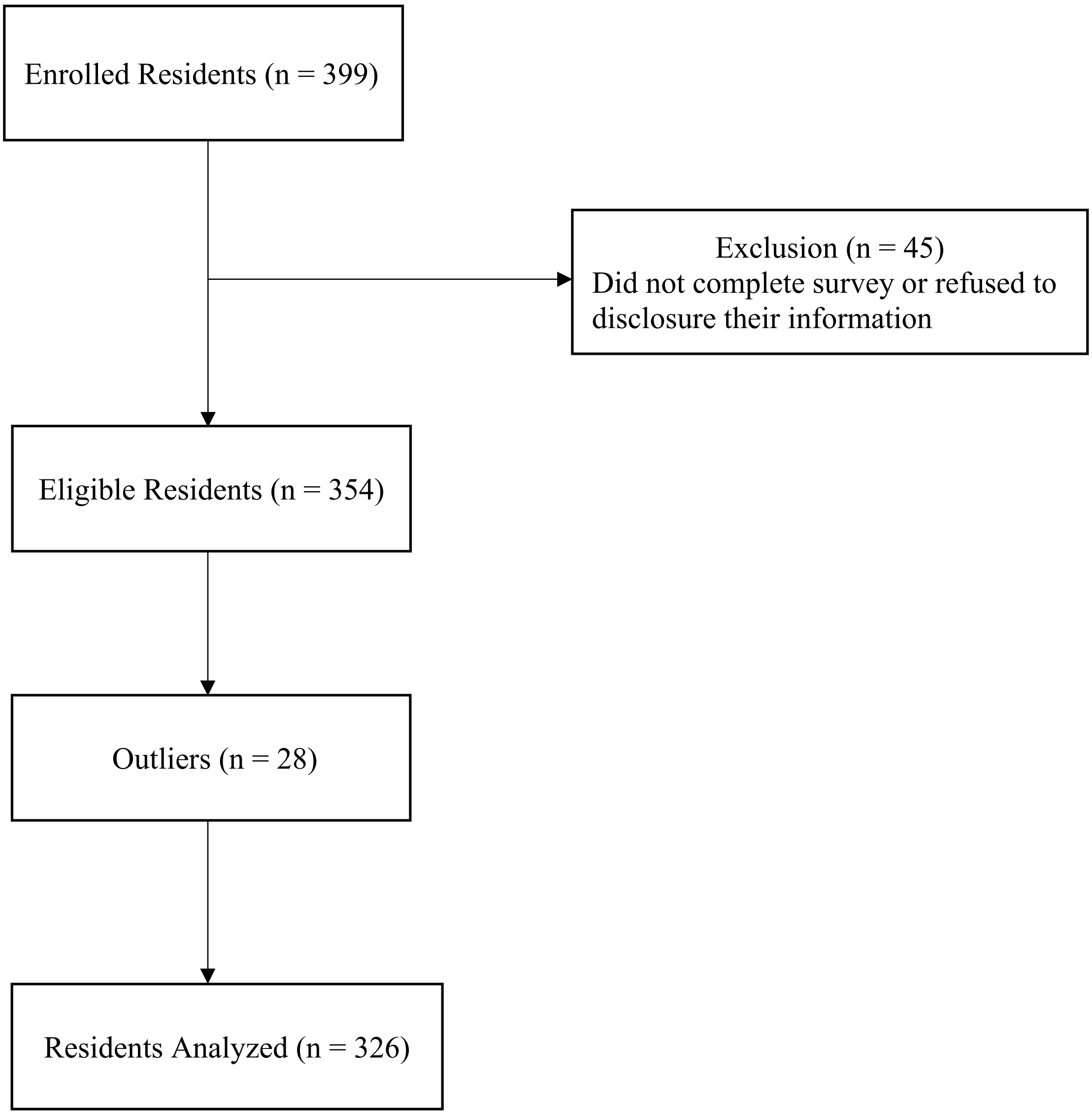

Supplemental Figure S1. Flowchart of patients' inclusion to this study

Supplemental Table S1. Descriptive Composite Seven Domains in Nursing Homes (n = 326).

Composite domain	Item	Weight	Mean (SD)
**Moving in**			-5.51 (0.73)
	First experience	-1.342	-
	Orientation help	-0.600	-
	Warm welcome	0.727	-
Composite moving in = (-1.342)*first experience + (-0.600)*orientation help + 0.727*warm welcome			
**Spending time**			21.78 (2.75)
	Time enjoyment	0.614	-
	Daily anticipation	-0.927	-
	Community connection	0.054	-
	Meaningful activities	2.974	-
	Outings events	0.050	-
	Activity preference	0.407	-
	Waiting time*	0.992	-
	Weekend activities	1.001	-
Composite spending time = 0.614*time enjoyment + (-0.927)*daily anticipation + 0.054*community connection + 2.974*meaningful activities + 0.050*outings events + 0.407*activity preference + 0.992*waiting time + 1.001*weekend activities			
**Care & services**			14.54 (2.16)
	Routine preferences	0.619	-
	Independence support	0.723	-
	Special therapies	0.682	-
	Goal setting	-0.571	-
	Goal achievement	0.861	-
	Therapy contact	0.864	-
Composite care & services = 0.619*routine preferences + 0.723*independence support + 0.682*special therapies + (-0.571)*goal setting + 0.861*goal achievement + 0.864*therapy contact			
**Caregivers**			27.62 (2.62)
	Staff knowledge	1.749	-
	Preference fulfillment	-0.377	-
	Frequent check-ins	1.344	-
	Promote independence	0.528	-
	Health engagement	0.541	-
	Response confidence	0.992	-
	Staff anger*	0.662	-
	Care explanation	0.284	-
	Consistent caregivers	0.314	
Composite caregivers = 1.749*staff knowledge + (-0.377)*preference fulfillment + 1.344*frequent check-ins + 0.528*promote independence + 0.541*health engagement + 0.992*response confidence + 0.662*staff anger + 0.284* care explanation + 0.314*consistent caregivers			
**Meals & dining**			15.44 (2.23)
	Favorite foods	-1.057	-
	Menu variety	1.177	-
	Food input	-0.241	-
	Food satisfaction	2.459	-
	Meal anticipation	0.912	-
Composite meals & dining = (-1.057)*favorite foods + 1.177*menu variety + (-0.241)*food input + 2.459*food satisfaction + 0.912* meal anticipation			
**Environment**			27.62 (2.44)
	Cleanliness level	-0.590	-

	Room navigation	0.833	-
	Outdoor access	-0.520	-
	Privacy level	2.550	-
	Alone time	0.880	-
	Item safety	-2.418	-
	Personal safety	4.915	-
Composite environment = (-0.590)*cleanliness level + 0.833*room navigation + (-0.520)*outdoor access + 2.550*privacy level + 0.880*alone time + (-2.418)* item safety + 4.915* personal safety			
**Facility culture**			32.07 (2.17)
	Speak up	-0.141	-
	Concern resolution	0.803	-
	Care involvement	0.047	-
	Staff happiness	0.790	-
	Extra effort	0.665	-
	Life inclusion	2.576	-
	Resident friendship	-0.034	-
	Recommend facility	1.932	-
Composite facility culture = (-0.141)*speak up + 0.803*concern resolution + 0.047*care involvement + 0.790*staff happiness + 0.665*extra effort + 2.576*life inclusion + (-0.034)*resident friendship + 1.932*recommend facility			

Footnote: SD = standard deviation. Weight for each item in each domain is given based on unstandardized beta weight by running general linear regression models.
